# Reduced Attention Allocation during Short Periods of Partially Automated Driving: An Event-Related Potentials Study

**DOI:** 10.3389/fnhum.2017.00537

**Published:** 2017-11-06

**Authors:** Ignacio Solís-Marcos, Alejandro Galvao-Carmona, Katja Kircher

**Affiliations:** ^1^Unit of Human Factors in the Transport System, Swedish National Road and Transport Research Institute (VTI), Linköping, Sweden; ^2^Department of Psychology, Universidad Loyola Andalucía, Seville, Spain; ^3^Institute of Biomedical Sciences, Universidad Autónoma de Chile, Santiago, Chile

**Keywords:** attention, mental fatigue, automated driving, underload, speed, event-related potentials, P3, N1

## Abstract

Research on partially automated driving has revealed relevant problems with driving performance, particularly when drivers’ intervention is required (e.g., take-over when automation fails). Mental fatigue has commonly been proposed to explain these effects after prolonged automated drives. However, performance problems have also been reported after just a few minutes of automated driving, indicating that other factors may also be involved. We hypothesize that, besides mental fatigue, an underload effect of partial automation may also affect driver attention. In this study, such potential effect was investigated during short periods of partially automated and manual driving and at different speeds. Subjective measures of mental demand and vigilance and performance to a secondary task (an auditory oddball task) were used to assess driver attention. Additionally, modulations of some specific attention-related event-related potentials (ERPs, N1 and P3 components) were investigated. The mental fatigue effects associated with the time on task were also evaluated by using the same measurements. Twenty participants drove in a fixed-base simulator while performing an auditory oddball task that elicited the ERPs. Six conditions were presented (5–6 min each) combining three speed levels (low, comfortable and high) and two automation levels (manual and partially automated). The results showed that, when driving partially automated, scores in subjective mental demand and P3 amplitudes were lower than in the manual conditions. Similarly, P3 amplitude and self-reported vigilance levels decreased with the time on task. Based on previous studies, these findings might reflect a reduction in drivers’ attention resource allocation, presumably due to the underload effects of partial automation and to the mental fatigue associated with the time on task. Particularly, such underload effects on attention could explain the performance decrements after short periods of automated driving reported in other studies. However, further studies are needed to investigate this relationship in partial automation and in other automation levels.

## Introduction

Fully automated vehicles (Level 5 in Society of Automotive Engineers (SAE’s) j3016 classification, see SAE, 2016) have received a great attention in recent years. However, expectations are that it will take some more time until they become a reality (Read, 2012). Currently, new advanced driver-assistance systems (ADAS) that enable the simultaneous longitudinal (i.e., speed and distance with the vehicle in front) and lateral control (i.e., position in the lane) of the vehicle, leading to partial automation or SAE’s Level 2 (SAE, 2016) are being commercialized. In Level 2 systems, the driver is relieved from using the pedals and, for short periods, from using the steering wheel. However, until higher automation levels are launched (e.g., Level 3 or “conditional automation”), the driver will still be required to closely monitor the system performance and intervene when necessary, as the system is not capable of handling every situation (e.g., degraded lane markings or visibility). This shift in the driver’s role may lead to changes in his/her behavior and performance that need to be investigated.

### Mental Workload and Resource Allocation in Automated Driving

One goal of vehicle automation is to reduce drivers’ mental demands, which may decrease the probability of human error and increase the drivers’ well-being and performance (Stanton and Marsden, 1996; Parasuraman and Riley, 1997). Reductions in mental demands during automated driving have been commonly reported in studies using different measurements (e.g., eye trackers, secondary tasks and physiological measurements. For a review see de Winter et al., 2014). Intuitively, these effects should make the driving task easier and improve the driving task itself.

However, the link between automated driving and increased safety does not seem to be that straightforward. In studies on partial or conditional automation, drivers have been observed to present slow reactions when prompted to resume control (for a review see Eriksson and Stanton, 2017). Other reported safety issues are decrements in driver situational awareness or inadequate mental models, (Saffarian et al., 2012). These effects suggest that, contrary to what expected, the lower demands in automated driving do not necessarily improve drivers’ attention, but even the opposite may occur. Gaining an understanding of this phenomenon is of great importance, particularly in automation levels in which drivers’ attention is continuously required (i.e., Level 2).

Different psychological mechanisms have been proposed as explanations for the aforementioned effects. One of which is complacency. Complacency occurs when drivers are confident that the system will handle every, or almost every, traffic situation, leading to a reduced allocation of resources to the automated tasks (Parasuraman and Manzey, 2010). Other studies have detected vigilance decrements due to mental fatigue during prolonged exposures to automated driving (Schmidt et al., 2009; Körber et al., 2015). For example, Saxby et al. (2008) observed that, over time, fully automated driving decreased the drivers’ task engagement level, particularly after 30 min of driving. This finding was interpreted as reflecting an effect of “passive” fatigue, a type of fatigue that arises when drivers are placed in a supervisory role for a prolonged time (Desmond and Hancock, 2001). According to Desmond and Hancock (2001), “passive fatigue” is different from “active fatigue”, which arises in sustained high demanding conditions. Similarly, Körber et al. (2015) also observed progressive vigilance decrements associated with “passive fatigue” during a partially automated drive on a highway.

Additionally, other studies have found performance problems after shorter periods, a concern that was already pointed out by Feldhütter et al. (2016). For example, it has been observed that after less than 5 min of automated driving, drivers presented a less controlled response to a potential collision (e.g., a lead car braking), as compared to when driving manually (Louw et al., 2015). Also, Cha (2003) observed that in 10-min long sessions of automated driving, drivers reacted slower to visual stimuli, presented a decreased skin conductance and blinked more often, which are indicators of drowsiness. In similar driving periods, Young and Stanton (2002) reported that, as the automation level increased, the drivers required longer glances to a visuomotor task to make a correct response (“attention ratio index”), which was interpreted as a lower attention allocation efficiency. The results were interpreted within the framework of their “malleable attentional resource theory” (MART) which predicts that the size of resource pool may transitorily change to accommodate the task demands. Particularly in automated driving, attentional capacity may have shrunk to accommodate to the low demands, thus reflecting an effect of underload. As highlighted by Young et al. (2014), underload needs to be distinguished from vigilance. In underload conditions, fewer resources would be allocated because either, attentional capacity has shrunk or, less effort is invested. Vigilance, however, is a highly demanding and stressful condition that requires operators to sustain attention over prolonged periods of time (Warm et al., 2008; Langner and Eickhoff, 2013). Different studies have reported higher perceived mental demands during vigilance tasks (Warm et al., 1996; Grier et al., 2003; Helton et al., 2005). A proposed explanation is that, resources deplete over time, leading to a reduction of available resources to cope with the vigilance task (Smit et al., 2004; Warm et al., 2008). This mechanism could explain the “passive fatigue” effects observed by Saxby et al. (2008) or Körber et al. (2015) during automated driving.

The evidence suggests that, besides the mental fatigue effects on attention after prolonged driving, driver attention allocation could also decrease with short exposures to automated driving. While mental fatigue is a well-studied topic in driving research, the mechanisms by which underload could affect attention remain to be investigated. Apart from Young and Stanton (2002), very few studies have explicitly attempted to detect attentional decrements in automated driving. One possible reason is that, as opposed to manual driving, attentional reductions in automated driving will rarely be reflected in any driving performance indicator (e.g., speed and lateral position variability; Campagne et al., 2004; Ting et al., 2008). Provided that the system keeps a constant speed and lateral position, there is a high risk that any decrement in attentional resources may go unnoticed until driver action is required, which may be too late. For this reason, other methods capable of detecting such attentional problems during automated driving should be applied. In this study, the event-related potentials (ERPs) technique was used to detect potential effects of automated driving on attention. Specific modulations of some attention-related ERPs may provide evidence that attentional processes are affected by short periods of partially automated driving, as suggested by Young and Stanton (2002).

### Event-Related Potentials as Indicators of Attention Allocation in Automated Driving

The ERPs consist of temporally separated components (e.g., P1, N1, P2, etc.) that represent brain responses to a specific event. Each component is defined by at least three parameters: (a) its polarity, positive (P) or negative (N); (b) its latency (usually measured in milliseconds), which shows “when” the brain responses occur (Kutas et al., 1977); and (c) its amplitude (usually represented in microvolts), which indicates the “intensity” of the processing or the “amount” of neural resources allocated (Polich, 2007).

Some components, such as frontal N1 (or N1) and P3, have be shown to be modulated by factors like task difficulty or mental fatigue. Typically, N1 has been associated with perceptual stages of information processing (Kramer et al., 1983; Kok, 1997), whereas P3 has been related to more central processes involved in the semantic processing of the information (Polich, 2007). Particularly, N1 and P3 amplitudes have been shown to be modulated by factors like vigilance decrements due to mental fatigue, which has been considered to reflect reductions in resource allocation (Polich, 2007). For example, Boksem et al. (2005) found changes in N1 and N2b amplitudes reflecting mental fatigue on both, top-down and bottom-up attentional processes. Uetake and Murata (2000) reported reductions in P3 amplitude with increases in mental fatigue in a visual computer task. Moreover, Martel et al. (2014) observed that attenuations in P3 amplitude could reliably anticipate attentional lapses in a vigilance task.

In driving research, ERPs have also been used to detect effects of vigilance decrements due to driving fatigue, alcohol and other factors. For example, Zhao et al. (2012) observed a significant decrement in P3 amplitude elicited by a visual oddball task after 90 min of driving, indicating an increased fatigue. Moreover, using a dual task paradigm, Schmidt et al. (2009), reported vigilance decrements after a 3-h of monotonous driving task. Vigilance was monitored by means of subjective questionnaires (i.e., Karolinska Sleepiness Scale, KSS; Åkerstedt and Gillberg, 1990), performance on an auditory secondary task (i.e., oddball task) and psychophysiological measurements (ERPs and heart rate). Vigilance decrements were shown by a linear increase in the “slow” reaction times and alpha spindle rate, as well as reductions in P3 amplitude and heart rate. In a similar setting, Wester et al. (2010) observed that higher levels of body alcohol concentrations were associated with worse driving performance, oddball task performance, as well as lower P3 amplitudes.

In this study, the ERP technique was used to evidence the potential underload effects of automated driving on drivers’ attention, suggested by Young and Stanton (2002). Complementarily, the mental fatigue effects on attention associated with the time on task were also explored. Specifically, modulations in N1 and P3 components, the most commonly reported, were analyzed to better investigate which specific attentional processes are affected by underload and mental fatigue.

### The Influence of Speed on Mental Workload and Attention Allocation

Further to the level of automation, another factor that influences drivers’ attention and performance is the vehicle speed. Speed is a well-known factor that contributes to drivers’ mental demand in manual driving (Cnossen et al., 2000; Patten et al., 2004). As speed increases, drivers have less time available to process the ongoing demands (e.g., surrounding cars, pedestrians, etc.), which is itself a source of cognitive load as stated by the Time-Based Resource Sharing Model (Barrouillet et al., 2004). The question remains whether such effects will also occur in partially automated driving. On the one hand, drivers’ monitoring demands in partial automation may be sensitive to speed changes, such that a greater effort needs to be invested at higher speeds. By contrast, drivers may as well trust the system to the extent that no extra effort is invested when speed increases. This would be the case of complacent drivers. In such case, drivers might feel confident to select higher speeds than when driving manually, as there is no perceived increase in risk or cognitive load. Despite the high relevance for traffic safety, the speed effects on drivers’ attention and the drivers’ speed preference in partially automated driving are poorly studied in the literature. For this reason, such effects will be investigated here.

### Research Questions and Hypotheses of the Study

Three main research questions (RQs) were established based on the gaps in knowledge identified above. These are presented below along with our hypotheses:

- RQ1: do drivers prefer different speeds when driving partially automated as compared to manually?
  ○ Hypothesis: we expect different mean speeds in the automated driving conditions.
- RQ2: does automation level (manual or partially automated) and/or speed level (low, high and comfortable) affect drivers’ subjective mental demand and vigilance, and objective attention allocation during short driving conditions?
  ○ Hypothesis: in line with previous studies (e.g., Young and Stanton, 2002), we expect lower perceived mental demands and lower resource allocation in the automated driving conditions, reflected in worse secondary task performance and lower amplitudes in N1 and P3. Also, we expect speed levels to affect mental demand and attention allocation in the manual driving conditions, but no effects when driving automated.
- RQ3: does the time on task (i.e., driving the different conditions) affect drivers’ subjective mental demand and vigilance, and objective attention allocation as measured by the ERPs?
  ○ Hypothesis: based on prior studies, we expect progressive increments in perceived mental demand and progressive decrements in vigilance, reflected in the subjective measurements and ERPs.

## Materials and Methods

### Participants

Twenty young adults (nine women) were recruited for this study. The mean age of the sample was 27.1 ± 3.8 years (ranging from 22 to 34 years). They had held their driving license for 7.2 ± 4.1 years on average. None of the participants had prior experience with automated driving.

All drivers reported a normal or corrected to normal visual acuity, and no history of neurological or psychiatric disease. Also, they were required to refrain from caffeine or tea intake for 4 h and alcohol for 24 h before the experiment day. The participants were recruited from the university population and rewarded with 500 SEK (approximately 60 US dollars) for 1.5–2 h of participation in the experiment.

This study was carried out in accordance with the recommendations of the American Psychological Association Code of Ethics with written informed consent from all subjects. All subjects gave written informed consent in accordance with the Declaration of Helsinki. The protocol was approved by the Regional Ethics Review Board in Linköping (Sweden).

### Subjective Measurements

Subjective mental demand was assessed by asking the participants to rate the question *“How mentally demanding was the task?”* (obtained from the “Mental demand” sub-scale in the NASA-TLX; Hart and Staveland, 1988) on a scale from 0 (low) to 20 (high), which was then transformed into a 0–100 scale. Additionally, as in Schmidt et al. (2009), the Karolinska Sleepiness Scale or KSS (Åkerstedt and Gillberg, 1990) was used as a subjective measure of vigilance. Drivers had to rate a scale ranging from 1 (extremely alert) to 9 (very sleepy). Subjective ratings for mental demand and vigilance were obtained immediately after each condition.

### Behavioral Measurements

#### Driving Task

**Figure 1:**
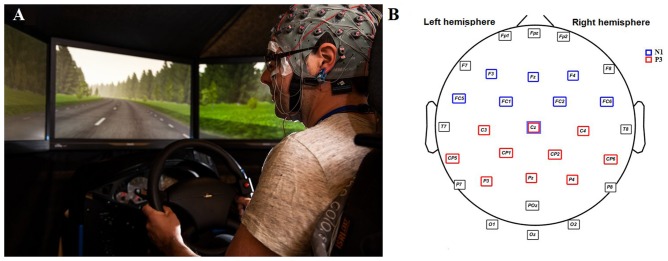
On the left **(A)**, a picture of one participant before the onset of the experiment. Explicit written consent for publishing this image was given by the participant. On the right side **(B)**, an illustration of the 30 scalp electrodes recorded and sets of electrodes analyzed for N1 (blue squares) and P3 (red squares).

The experiment was conducted in a fixed-base simulator comprised of a car seat, steering wheel, automatic gearbox and dashboard (see Figure 1). The scenarios were presented through five screens covering the 190° field of view and consisted of a rural road with two lanes, one for each direction. No other vehicles were present in the participants’ lane, however, low density oncoming traffic was present.

The participants were told that, in some conditions, they were going to drive a partially automated car that was not fully reliable, therefore they should always monitor its performance. To increase such uncertainty, a road was designed such that straight stretches were alternated with mild right and left curves (radius = 1000 m). Although the automated lane positioning was rather stable most of the time, some occasional slight lane excursions may occur. In such cases, the participant could either wait for the system to reposition the car in the lane, or manually correct it by using the steering wheel.

The participants drove the same scenario six times consecutively under six different conditions. The different conditions consisted of the combination of two automation levels and three speed levels. The factor *automation level* was comprised of: (a) manual driving (the participant controlled the lateral and longitudinal position of the vehicle); and (b) automated driving (the longitudinal and lateral controls were automated). The factor *speed* consisted of three levels: “low” (70 km/h), “high” (120 km/h) and “comfortable speed”. Each condition lasted approximately 5 min.

#### Secondary Task: Auditory Oddball Task

As in previous studies (Schmidt et al., 2009; Wester et al., 2010; Körber et al., 2015), a dual-task paradigm was used in which the participants were instructed to perform an auditory oddball task while driving in the different conditions. This task was used to elicit the ERPs and account for potential performance decrements associated with attentional reductions. In each condition, a random sequence of high pitch (“target tone”, 1200 Hz) and low pitch (“standard tone”, 800 Hz) tones, was presented at fixed intervals of 1 s through two speakers at 70 dB (5 ms r/f). Each tone was presented for 50 ms and the probability was 80% for the standard and 20% for the target tones. The participants were instructed to respond as quickly and accurately as possible to the target tones by pressing a button attached to the steering wheel (Figure 1A).

### Psychophysiological Measurements: ERPs

Electrical brain activity was continuously recorded from 30 scalp Ag-AgCl active electrodes distributed according to the 10–20 international system and referenced to right and left earlobes. Horizontal and vertical ocular electrodes (HEOG and VEOG, respectively) were placed on the outer left and right canthi and above and below the left eye. Impedance levels were kept below 10 kOhms. A G.Tec amplifier was used for the brain signal recordings (G.Tec Medical Engineering).

EEG data analysis was performed in Matlab R2014b using EEGlab 13.4.4b, an open source toolbox developed by Delorme and Makeig (2004). The EEG signal was digitized with a sampling rate of 500 Hz and amplified in the range between 0 kHz and 2.4 kHz. Data were offline resampled at 256 Hz and bandpass-filtered between 0.1 Hz and 20 Hz. ERP averages were calculated from 100 ms prior to 800 ms after the stimulus presentation. All ERP averages were corrected to the baseline over the pre-stimulus interval (−100 ms to 0 ms). Artifacts related to muscle, blinks and ocular movements were minimized by using the “runica” ICA algorithm (Lee et al., 1999). Moreover, trials where amplitude exceeded ±75 μV on HEOG were discarded.

The N1 and P3 components elicited by the target tones from the oddball task were analyzed given their shown sensitivity to attention resource allocation (Kramer and Spinks, 1991; Polich, 2007). These two components were subtracted from each participant following the guidelines from Duncan et al. (2009). The maximum peaks for N1 and P3 were searched on a wide range of frontocentral and centroparietal electrodes, respectively (see Figure 1B). Thus, we ensured that only the maximum peaks from each participant were included in the analyses. N1 component was identified as a negative deflection with its maximum peak occurring between 75 ms and 150 ms after the stimulus in frontocentral electrodes (blue squares). P3 component was classified as the largest positive peak amplitude in a time window between 250 ms and 400 ms occurring in parietocentral regions (red squares). The electrodes used to find N1 and P3 were selected according to the topographical distributions of such components typically reported in the literature (Alcaini et al., 1994; Friedman et al., 1997).

### Experimental Design

**Figure 2:**
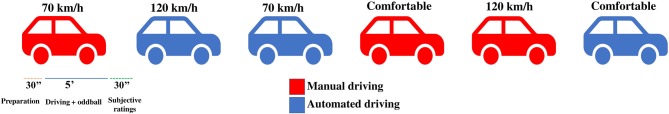
An example of a sequence of conditions in the experiment. The actual order was randomized for each participant. Each condition consisted of 30 s of just driving (preparation), followed by 5 min of driving and oddball performance. Subjective ratings of mental demand and vigilance were performed right afterwards.

A 2 × 3 factorial within-subject design was used to analyze the effects of *automation level* (manual and partially automated) and *speed* (low, comfortable and high). The order of the six driving conditions was randomized across participants with the constraint of not presenting three automated driving conditions consecutively. This way, we tried to control for the vigilance decrements associated with exposures to automated driving longer than 10 min (Saxby et al., 2008; see Figure 2).

As dependent variables, measurements were obtained from the driving simulator, subjective questionnaires, oddball task performance and brain recordings. From the driving task, average speed was obtained to account for drivers’ preferences in the “comfortable speed” conditions. Performance on the oddball task was assessed by logging the correct responses and reaction times to the target tones. Additionally, given the few number of misses and false alarms observed, a global “inaccuracy index” was calculated by adding both indexes. Finally, from the psychophysiological measurements, amplitudes and latencies of P3 and N1 were compared across all the conditions.

A *post hoc* power analysis conducted on G.Power 3.1.9.2 (Faul et al., 2007) indicated that, with a sample size of 20 participants, α and β levels of 0.05 and a correlation between repeated measurements of 0.5, the statistical power was >0.9, which is adequate to detect large effect sizes ($\eta_{\text{p}}^{2}$ > 0.14).

### Procedure

The present study took place in VTI facilities in Linköping, Sweden. Upon arrival, the participants were briefed about the purposes of the experiment. Then the participants signed an informed consent sheet and completed questionnaires with general information. Next, they were given practice in the oddball task and the simulated manual and partially automated driving. After the completion of the training (approximately 15 min), the physiological equipment was set on the participants (an G.Tec elastic cap consisting of 30 active electrodes and four ocular electrodes, G.Tec Medical Engineering). Then, the actual experiment started consisting of six consecutive drives at different speeds and automation levels. Prior to the onset of each drive, the participants drove for 30 s without the oddball task to acclimatize to the driving conditions. Then, a 5-min period of driving and oddball task started. After each condition, the participants were asked to report their perceived mental demand (“Mental demand” sub-scale from NASA-TLX) and vigilance level (KSS; see Figure 2).

In the manual driving conditions, the drivers had to maintain the required low (70 km/h) or high (120 km/h) speeds throughout the whole drive. In the “low” and “high speed” conditions, the participants were told that it was fully acceptable to let the speed vary a bit around the target speed, thus preventing a potential increase in mental effort from focusing unnaturally hard on maintaining the required speed very accurately. In the “comfortable speed” condition, drivers were allowed to modify the speed at all times.

In the automated driving conditions, the low and high speeds were constant from the beginning. In the “comfortable speed” condition, the participants started driving manually until they found a comfortable speed. Then he/she communicated this to the experimenter who manually set the speed. This was done during the acclimatization driving period (the first 30 s, see Figure 2). In the automated conditions, the speed was controlled by the system and the drivers could not change it.

Once all the conditions were completed, the physiology equipment was removed and the participants were thanked for their participation.

### Statistical Analyses

Means and standard errors (SE) for each dependent variable in each condition were calculated. Parametricity of the data was confirmed by using the Shapiro-Wilk’s test in all the dependent variables and in all conditions, except for the accuracy and inaccuracy indices.

Comparisons between comfortable speeds in the automated and manual driving conditions were performed by using a two-tailed paired *t* test. In addition, a correlation analysis was conducted to check whether drivers’ speed preferences were consistent across automation levels. Moreover, two-way repeated measures analysis of variance (ANOVAs) was conducted for each dependent variable to account for main and interaction effects of *speed* and *automation level*. Sidak’s pairwise comparisons were used to correct for type-I error.

To analyze the effects of mental fatigue associated with the time on task, all conditions were re-arranged according to the order in which they were presented to each participant. Thus, six new variables were created (i.e., first condition, second condition, third condition and so on). A chi-square test (*χ*^2^) confirmed that automation level conditions were evenly distributed over time ($\chi_{\left(5\right)}^{2}$ = 0.96, *P* > 0.05). Thus, changes in our dependent variables could be more confidently attributed to time on task, rather than to other effects such as an overrepresentation of automated conditions in the last part of the experiment. A one-way repeated measures ANOVA was used to examine the time on task effect on each dependent variable. When significant, pairwise comparisons were conducted between the first and last sessions. In addition, trend analyses were performed by using orthogonal polynomial contrasts.

All effects are reported as significant at *P* < 0.05. Partial eta-squared ($\eta_{\text{p}}^{2}$) was calculated as a measure of relative effect size.

## Results

This section is subdivided into different parts, according to our RQs. In the first part, comparisons between driver’s preferences of speed in automated and manual driving were conducted. In the second part, analyses were performed to account for the effects of *automation level* and/or *speed* on mental demand and attention allocation measurements. Finally, in the last section, the “time on task” effects on mental demand and attention allocation, and the trend analyses are presented.

### RQ1: Do Drivers Prefer Different Speeds When Driving Partially Automated as Compared to Manually?

**Figure 3:**
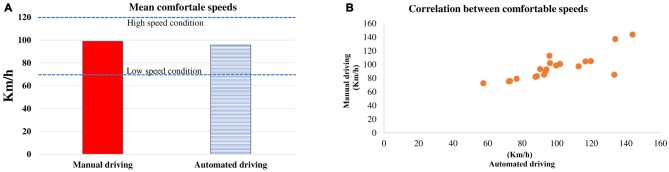
Mean (and standard error, SE) comfortable speeds selected in the manual and automated driving conditions **(A)**. Blue lines indicate the speeds required in the high speed conditions and low speed conditions. On the right side **(B)**, a scatter plot is presented with the mean comfortable speeds per participant in the manual and automated conditions.

The Figure 3A shows the average speeds (and SEs) in the comfortable speed conditions in both automation levels. In both cases, participants preferred speeds that were between those required in the high speed (120 km/h) and low speed conditions (70 km/h; Manual driving = 99.02 ± 1.64; Automated driving = 95.7 ± 1.41). On average, similar speeds were preferred indicating no effect of *automation level* (*t*_(19)_ = 0.55, *P* > 0.05). A significant correlation analysis showed a strong linear association between the preferred speeds in both automation levels (*r* = 0.89, *p* < 0.01; see Figure 3B).

### RQ2: Does Automation and/or Speed Levels Affect Drivers’ Subjective Mental Demand and Vigilance, and Attention Allocation?

#### Subjective Measurements

**Figure 4:**
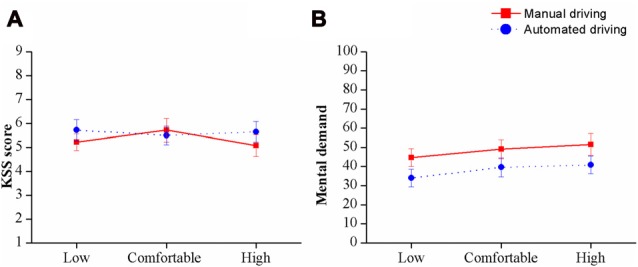
Mean scores (and SE) on the Karolinska Sleepiness Scale (KSS) **(A)** and mental demand **(B)** in each condition.

Mean scores and SEs on subjective mental demand (Sub-scale “Mental demand” from NASA-TLX) and vigilance scales (KSS) are presented together in Figure 4. Significant main effects of *speed* (*F*_(2,38)_ = 4.61, *P* < 0.01, $\eta_{\text{p}}^{2}$ = 0.2) and *automation level* (*F*_(1,38)_ = 7.8, *P* < 0.01, $\eta_{\text{p}}^{2}$ = 0.29) on subjective mental demand were found. Globally, the automated driving conditions were perceived as less mentally demanding than the manual driving conditions. Regarding the main effect of *speed*, pairwise comparisons revealed lower scores in the low speed than in the high speed conditions (*p* < 0.01). Contrary to our hypothesis, no interactions were found between *speed* and *automation level* (*F*_(2,38)_ = 0.22, P = n.s., $\eta_{\text{p}}^{2}$ = 0.01).

Additionally, the analyses conducted on the KSS scores reflected no effects of *speed* and/or *automation level* on the participants’ subjective vigilance. Mean scores ranged between 5 (neither alert nor sleepy) and 6 (some signs of sleepiness) in all conditions.

#### Behavioral Performance on Oddball Task

Table 1 displays the scores on each oddball index. Overall, the participants performed well on the oddball tasks, as reflected by the high percentage of correct responses (over 98% in all conditions), the low scores in the inaccuracy index and the similar reaction times across conditions. No main or interaction effects were observed.

**Table 1:** Scores in the oddball task in each condition.

Dependent variables	Manual driving			Automated driving		
	Low	High	Comfortable	Low	High	Comfortable
Accuracy	98.87 (0.38)	99 (0.33)	99.37 (0.24)	99.5 (0.23)	98.87 (0.33)	98.75 (0.38)
Inaccuracy	0.65 (0.16)	0.45 (0.13)	0.4 (0.15)	0.6 (0.18)	0.75 (0.22)	0.6 (0.18)
Reaction times	366 (9.5)	363 (7.44)	366 (9.04)	364 (9)	377 (11.1)	363 (10.09)

*Means and SEs (in brackets) are presented. Reaction Times in Milliseconds are shown*.

#### Psychophysiological Measurements: ERPs

##### N1 component

**Figure 5:**
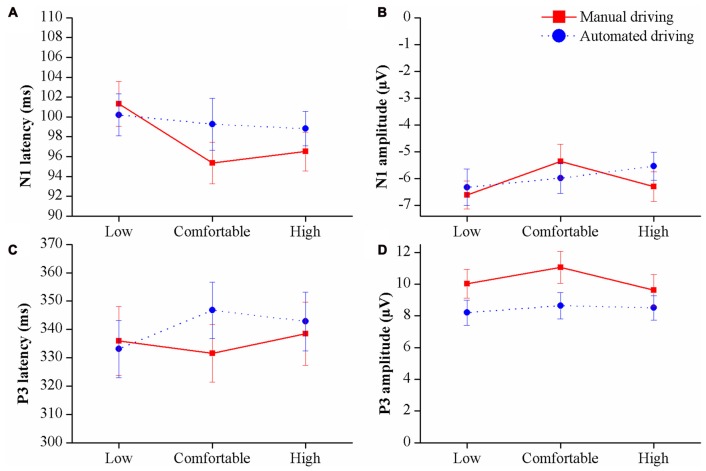
N1 **(A,B)** and P3 **(C,D)** latencies and amplitudes in the different driving conditions. Means and SEs are shown.

Figures 5A,B display N1 amplitudes and latencies observed in each condition. Statistical analyses on N1 amplitude and latency did not reveal any significant effects of *speed* and/or *automation level*, nor any interactions.

##### P3 component

**Figure 6:**
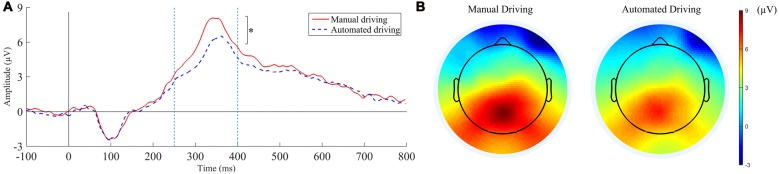
P3 component grand average waveforms **(A)** and topographical distribution **(B)** in the manual and automated driving conditions. The time window used for the topographical representation was 270–400 ms after the stimulus onset. *P < 0.05.

P3 mean amplitudes and latencies for each condition are represented in Figures 5C,D. A main effect of *automation level* was observed on P3 amplitude (*F*_(1,38)_ = 5.181, *P* < 0.05, $\eta_{\text{p}}^{2}$ = 0.245). Grand average waveforms and topographical distribution are presented in Figure 6 to illustrate such main effect. P3 latency was not influenced by any of the independent factors.

### RQ3: Does the Time on Task Affect Drivers’ Subjective Mental Demand and Vigilance, and Objective Attention Allocation?

**Figure 7:**
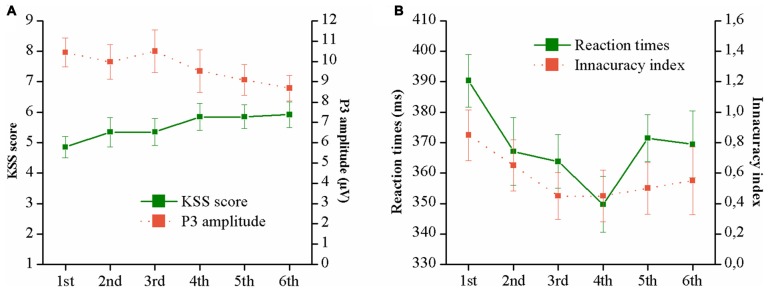
P3 amplitudes, KSS scores **(A)**, reaction times and inaccuracy index **(B)** across the different conditions over time.

The results of the ANOVAs for the time on task effects are presented in Table 2. Significant effects were observed on KSS scores, P3 amplitudes and reaction times. *Post hoc* comparisons indicated significant differences between the first and last conditions for the KSS scores and P3 amplitudes (KSS: *P* < 0.01; P3 amplitude: *P* < 0.01). As for reaction time, a significant *post hoc* was found when comparing the first and fourth conditions (*P* < 0.05). Also, significant trends were found for these variables. Regarding KSS, a linear trend was observed showing an increment in the scores over time (see Figure 7A). Similarly, P3 amplitudes linearly decreased across the different conditions (see Figure 7A). As shown in Figure 7B, a quadratic trend was observed for the variable *reaction time*. Reaction times progressively decreased until the fourth condition (that is, approximately 20 min after the experiment onset), then, an increase is observed in the last two drives. Finally, a quadratic trend was observed for the *innacuracy index*, although it did not reach the significance level (*P* = 0.07). As shown, in Figure 7B, the number of misses and false alarms decreased progressively until the 3rd condition, then, slight increases were detected.

**Table 2:** Time on task effects and trend analyses for each dependent variable.

Dependent variables	Time on task effects			Trend analyses			
	*F*_(5,95)_	*P*	$\eta_{\text{p}}^{2}$	Type	*F*_(1,19)_	*P*	$\eta_{\text{p}}^{2}$
Mental demand	1.33		0.07				
KSS	5.12	**	0.28	Linear	11.39	**	0.47
Accuracy	0.32		0.02				
Innacuracy	1.1		0.05				
Reaction timeª	4.1	*	0.15	Quadratic	5.98	*	0.26
P3 amplitude	4.67	*	0.16	Linear	6.94	**	0.3
P3 latency	0.98		0.05				
N1 amplitude	2.01		0.11				
N1 latency	0.63		0.04				

^a^Degrees of freedom were adjusted to F_(3.46,58.89)_ by using Greenhouse-Geisser’s correction. *P < 0.05, **P < 0.01.

## Discussion

Our findings suggest that the automation level (manual or partially automated), does not have any influence on drivers’ preference of speed, nor on the speed level effects on the drivers’ mental demand and attention. However, we found that the automation level did affect the drivers’ attention, as shown by modulations in P3 component. Particularly, decrements in P3 amplitude were observed that may indicate reductions in drivers’ attention allocation during automated conditions. These findings would support our hypothesis inspired in Young and Stanton (2002) findings that driver attention may be affected by an underload effect after just a short period of automated driving. Also, they provide support for the use of ERP technique in the detection of attentional problems during automated driving. Next, the results are discussed per RQ.

### Preferences of Speed in Manual and Partially Automated Driving (RQ1)

Contrary to our assumption, the drivers selected similar speeds in manual and automated driving during the “comfortable speed” conditions. It is possible that the preference for speed is not influenced by the level of automation indicating that drivers would prefer the system to behave like themselves. However, other potential reasons for the lack of difference found should be accounted. One potential explanation is that, in the automated driving condition, the comfortable speed set at the beginning could not be changed later on by, for example, using specific buttons attached to the steering wheel (as in commercial vehicles). We considered that the inclusion of such option could have interfered with the performance of the oddball task, which also required a button response, thus limiting the interpretation of our results. However, this may have also prevented us from observing possible increases in speed during the drive. Another reason could be that, since none of the drivers had previous experience with partially automated driving, they selected “comfortable” speeds based on their experience with manual driving. In this regard, a positive correlation analysis confirmed that there were intra-individual consistencies in the preferred speeds across automation levels.

Whether drivers will prefer to drive faster in future automated vehicles is of great importance from a traffic safety perspective. While it is possible that such differences do not really exist, this should be further investigated in other traffic situations such as straight highways. We also encourage future studies to analyze drivers’ preference of speed under higher automation levels as well (e.g., Level 3 or “conditional automation”). Also, it is highly recommended to include drivers with prior experience with real or simulated automated vehicles.

### Speed and Automation Level Effects (RQ2)

In line with the literature, automated driving and low speeds were perceived as mentally less demanding by the participants. In other studies, reductions in the primary task demands (e.g., a videogame) have led to larger amplitudes in certain ERPs components elicited by a secondary task (e.g., oddball task), showing more resources available (Ullsperger et al., 2001; Allison and Polich, 2008). However, as we hypothesized based on previous studies on automation (e.g., Young and Stanton, 2002; Louw et al., 2015), the opposite effect was observed here. P3 amplitudes were lower in the partially automated driving conditions as compared to manual driving conditions, which would indicate that fewer “neural” resources were allocated for the categorization of the tones (Polich, 2007). Despite this, the oddball task performance was not affected, which is consistent with other studies using this task (Körber et al., 2015). Similarly, no decrements were directly observed by Young and Stanton (2002) in their visuomotor secondary task either. Only when the number of correct responses was compared with the time spent glancing at the task (i.e., “attention ratio”), a lower attentional efficiency was found. Regarding the variable *speed*, it had no effects on any of the subjective, behavioral or psychophysiological measurements of attention.

The lack of differences in the performance of the oddball task could show that the participants were performing at ceiling. In fact, as stated by de Waard (1996), performance indicators may not be sensitive to low-to-moderate demands and, therefore, other more sensitive techniques should be used. In this sense, analyzing the ERPs elicited by the auditory oddball tasks might be a good solution, in the light of our results. Still, the link between the observed decrements in P3 amplitude and its real implications on driving performance need to be better investigated. Future studies combining ERP measures with take-over situations may help to better understand the relationship between the driver attentional state and his/her capacity to resume control.

From a methodological perspective, our results indicate that the ERP technique might be a valuable tool to better detect attentional effects of automated driving, as many of the driving performance indicators are no longer useful. Nevertheless, the application of the ERP technique necessarily requires the control of other variables that could limit their interpretation. As Polich and Kok (1995) indicated, P3 amplitude could be as well affected by factors such as sleep hours, caffeine and alcohol consumption or motivation. While some of these variables were to some extent controlled in this work, the influence of other variables, such as the drivers’ motivation towards the experiment, should not be discarded.

From a more conceptual point of view, the interpretation of our results may be more challenging. On the one hand, it seems unlikely that the lower P3 amplitudes in the automated conditions indicate vigilance decrements associated with mental fatigue, in part because the experiment was designed to mitigate as much as possible such effects (e.g., short driving conditions in a randomized order). Also, the subjective vigilance scores were similar throughout all the conditions and they were never high (KSS scores ranged from 5 to 6). Then, our results could be explained by MART (Young and Stanton, 2002). According to this, the drivers’ attentional capacity may have “shrunk” as an effect of underload, leading to fewer resources available. However, we cannot confirm that such hypothetical effect decreased the driver’s attentional efficiency, following the authors’ interpretation. To assert that, we should have seen decrements in the performance of the secondary task, which we did not find. The observed lower P3 amplitudes in the automated conditions may as well explained by the effort-regulation hypothesis (Hancock and Warm, 1989). According to this, less effort (or resources) was invested when the overall demands decreased. In our study, such “shrinkage” in attentional capacity or lower effort investment, may have particularly affected the allocation of resources for the semantic processing of the tones (represented by P3), but not for their perceptual processing (represented by N1). Such dissociation may be also explained by the fact that N1 effects require more time to develop (Näätänen, 1992), consequently, they were not observed in our short conditions.

Regardless of the underlying mechanisms, these findings could reflect a generalized effect of underload on driver attention during partial automation. Underload would affect drivers’ ability to allocate attention not only to the driving task but also to other ongoing tasks. In our study, fewer resources were allocated to the oddball task in the automated conditions, despite the driving task being less demanding and the drivers being instructed to perform it as good as possible. From a safety perspective, this may have relevant implications when the drivers are required to resume control, particularly in critical situations. In such conditions, drivers may need some time to recruit the neural resources necessary to perceive and comprehend the situation, and react accordingly. This could be a psychophysiological mechanism underlying the safety issues reported in automated driving, such as slower take-over reactions (e.g., Eriksson and Stanton, 2017).

### Time-on-Task Effects (RQ 3)

We observed that driving time affected the different subjective, cognitive and psychophysiological measurements. KSS linearly increased and P3 amplitude linearly decreased over time, which is consistent with other studies using prolonged driving periods (Schmidt et al., 2009; Zhao et al., 2012) or continuous performance of cognitive tasks (Uetake and Murata, 2000; Martel et al., 2014). These effects should show progressive decrements in the ability to sustain attention due to mental fatigue. Despite this, no increments in subjective mental demand were observed as in other studies (Warm et al., 1996; Grier et al., 2003; Helton et al., 2005). Paradoxically, a different pattern was observed in the oddball task. Progressive improvements were seen, at least until 20 min after the experiment onset (i.e., the 4th condition), when performance stabilized showing similar or, even, slight decrements in the ensuing conditions. The inclusion of longer or more experimental conditions would have probably contributed to better determine whether and to what extent, performance could be affected. Based on our findings, we could only hypothesize that, as more practice was gained throughout the conditions, more automatic processes were involved in the performance of the tasks, resulting in better performances with fewer attentional resources invested. However, as the task continued, such automatic processes might have also been affected, leading to a greater task disengagement. While this is not so clear in our results, previous studies have reported findings supporting this (Saxby et al., 2008; Schmidt et al., 2009).

In general, the results presented above may be indicative of an increased mental fatigue over time. However, the underlying mechanisms of the development of mental fatigue remain unclear. Based on the resource theories, it is possible that a progressive depletion of the attentional resources may have occurred (Grier et al., 2003; Helton and Warm, 2008). As a result, fewer resources were available to perform the oddball task over time, reflected in P3 amplitude decrements. Alternatively, according to other authors (Lorist et al., 2009; Gergelyfi et al., 2015), such mental fatigue effects may have been mediated by a motivation loss over the course of our experiment. As stated by Tops et al. (2004), when the invested effort is not proportionally rewarded, the motivation to keep engaged in the task may decay leading to mental fatigue. Given that in our study the participants’ motivation was not assessed or controlled by rewarding participants for their performance, this explanation should not be discarded.

#### Summary and Recommendations

Our findings suggest that different factors may affect driver attention during a partially automated driving journey. One relates to the underload effects of automated driving. When placed in a supervisory role, drivers may allocate fewer resources due to a shrunk attentional capacity (Young and Stanton, 2002) or less effort invested (Hancock and Warm, 1989). This effect starts as early as 5 min of automated driving and may require specific measurements of information processing, such as ERPs, to be detected. The other mechanism relates to the mental fatigue associated with prolonged driving, which occurs regardless of the automation level. Such effect seems to be more easily detected by a wider range of measurements (i.e., subjective, behavioral and ERPs).

To analyze the safety impacts of these two factors, it would be interesting to include scenarios in which drivers are requested to take over control after different periods of automated driving. The analysis of such reactions, along with specific measurements of attentional processing (e.g., ERPs), can contribute to shedding more light on the short and long-term effects of automated driving on driver attention, performance and safety. Moreover, the analysis of other ERP components sensitive to mental fatigue (e.g., Boksem et al., 2006; Lorist, 2008) would help to explore in depth the specific attentional processes affected in automated driving.

#### Current and Future Implications

The results presented here are of interest for the evaluation and design of future automated systems. First, they indicate that attentional reductions may be detected by using specific techniques such as ERPs. This is particularly relevant to evaluate the effects of current and future automated systems. Second, different design solutions or countermeasures may be needed for the specific effects of automation and mental fatigue on drivers’ attention. For the automation-specific effects (i.e., short-term effects), driver-vehicle interactions should be designed ensuring that the “right” tasks are being automated while avoiding placing the driver in a “too” passive supervisory role. Regarding the mental fatigue effects during automated driving, some driver attentional state monitoring systems may be developed based on different parameters such as eye movements or body position. Whenever the system detects potential symptoms of mental fatigue, specific operations can be performed, such as giving back control to the driver or suggesting nearby places where the driver can take a break.

To our knowledge, this is the first study using ERPs in automated driving. This could be a promising approach that could greatly contribute to understanding the attentional and performance effects of automated driving and also in other contexts where automation is present (e.g., aviation or nuclear plants). Therefore, future studies investigating the applications of this method in dynamic and ecological contexts, such as automated driving are necessary.

## Conclusion

As long as automated driving requires the driver supervision (as in Level 2 or partial automation), some potential problems may occur that could affect safety. In this study, drivers’ attention was observed to be affected by different factors. One of them can start at early stages of automated driving, and it is a consequence of the perceived low demand itself. As suggested elsewhere, this could be explained by a shrinkage in drivers’ attentional capacity, or a lower effort invested. The other effect occurs after a prolonged time of driving, regardless the level of automation. Such effect reflects vigilance decrements associated with the increasing mental fatigue over time. Given that, in automated driving, drivers’ attentional decrements cannot be detected through the increased lateral position and speed variabilities, other measurements were needed. As shown here, ERPs may be an adequate solution, not only to detect decrements in attention allocation, but also, to better investigate the attentional processes that are affected during an automated driving journey. To our knowledge, this is the first study using ERPs to investigate the attention-related safety issues reported in the literature of automated driving. Further research on its applications in automated driving or other contexts where automation is present, is encouraged. The use of ERPs may be considered during the evaluation and design of more human-centered systems.

## Author Contributions

IS-M and KK: conceptualization and design of work; IS-M: data acquisition and analyses, writing; IS-M, AG-C and KK: results interpretation, final approval; AG-C and KK: critical revision, supervision; KK: funding acquisition.

## Conflict of Interest Statement

The authors declare that the research was conducted in the absence of any commercial or financial relationships that could be construed as a potential conflict of interest.

## Abbreviations

ADAS: Advanced Driver Assistance Systems
ERPs: Event-related potentials
KSS: Karolinska Sleepiness Scale
MART: Malleable attentional resource theory
RQ: Research Question
SAE: Society of Automotive Engineers.
